# Hypergravity enhances the therapeutic effect of dexamethasone in allergic asthma and rhinitis animal model

**DOI:** 10.1371/journal.pone.0197594

**Published:** 2018-05-17

**Authors:** Tae Young Jang, Ah-Yeoun Jung, Soonjo Kwon, Young Hyo Kim

**Affiliations:** 1 Department of Otorhinolaryngology, Head and Neck Surgery, Inha University College of Medicine, Incheon, Republic of Korea; 2 Department of Biological Engineering, Inha University, Incheon, Republic of Korea; South Texas Veterans Health Care System, UNITED STATES

## Abstract

We investigated whether the therapeutic effects of dexamethasone for allergic asthma and rhinitis were enhanced in mice when exposed to hypergravity. Forty mice were divided into 5 groups (n = 8/group): Control group received saline intraperitoneally (i.p.) and intranasally (i.n.); Asthma group received i.p./i.n. ovalbumin (OVA) for inducing allergic asthma/rhinitis; Dexa group received i.n. dexamethasone (0.75 mg/kg) 30 minutes before each OVA challenge; Hypergravity group was subjected to allergic asthma/rhinitis as well as exposed to 5 G hypergravity for 30 days; Finally in Dexa/Hypergravity group, hypergravity and dexamethasone were used simultaneously during induction of allergic asthma/rhinitis.

Dexa group and Hypergravity group showed a significant decrease in serum total IgE levels compared to the Asthma group (*p*<0.05). Dexa/Hypergravity group showed greater IgE decrease compared with Dexa group (*p* = 0.040). Compared with the monotherapy groups, Dexa/Hypergravity group showed significantly fewer eosinophils in BAL fluid (*p*<0.05). Dexa/Hypergravity group showed significantly decreased eosinophilic infiltration into the lungs and nasal cavity (*p*<0.05). *EC-SOD* (extracellular superoxide dismutase) expression was significantly upregulated in the Hypergravity group and Dexa/Hypergravity group, compared with the Dexa group (*p*<0.05). In conclusion, hypergravity enhanced the therapeutic effect of dexamethasone in a murine model of allergic asthma and rhinitis. Therefore, combination could be a promising strategy, and one of its mechanisms could be up-regulation of *EC-SOD* expression.

## Introduction

We define “hypergravity” as the gravity of a magnitude larger than that of the Earth’s gravity, the planet on which we live. Hypergravity of a considerably large magnitude adversely affects the body, and can even lead to death. However, according to recent research results, hypergravity of an appropriate magnitude has been proposed to have a positive effect on living organisms.[1] We could define this phenomenon as a “hormetic effect,” which has a positive effect at the appropriate magnitude, but has a negative effect at an exceedingly large magnitude.[2] In fact, Minois and researchers suggested that an appropriate degree of gravity had the effect of extending the life span of the *Drosophila*.[1]

To investigate the effect of hypergravity on the living organisms, many researchers mainly used centrifugal force. In short, it is a method to increase the gravitational acceleration by the centrifugal force by rotating a gondola on which an animal or a human is mounted. Using these instruments, they evaluated changes inside the body after up to several weeks of exposure to hypergravity.[3–6] It is quite well known that the immune system is one of the most affected biological systems during the space travel. As astronauts performed space missions for a long time, their immunological dysfunctions became one of their main health problems.[7]

On the other hand, there are relatively so few studies on the effects of hypergravity on the immune system. Guéguinou et al. investigated the changes in IgG, IgM, and IgA immunoglobulins in experimental animals exposed to hypergravity up to 3G for three weeks.[8] Voss et al. also studied the changes in humoral immunity after short-term space flight.[9] Therefore, we could guess that in cases of diseases associated with allergic immunity, such as allergic asthma or rhinitis, there is a possibility that the course of the disease may be changed by increased gravitational stimuli. However, there have been no studies on the relationship between hypergravity and allergic diseases.

In our previous studies, we have shown that an appropriate degree of hypergravity could produce a hormetic effect on animals with allergic diseases.[10] However, whether this hormesis due to hypergravity is efficacious enough to be comparable to actual drug therapy has not been studied yet. Furthermore, no studies have been conducted to determine whether the anti-allergic effect of the combination of hypergravity and drug therapy on experimental animals with allergic diseases is greater than that of medication alone.

Therefore, we aimed to compare the efficacy of the treatments with dexamethasone, hypergravity, and the combination of dexamethasone and hypergravity in mice with allergic asthma and rhinitis. For this purpose, we observed and evaluated the following indicators: (i) the serum levels of total IgE and ovalbumin (OVA)-specific IgE, (ii) the number of differential inflammatory cells, such as eosinophils, neutrophils, and lymphocytes in bronchoalveolar lavage (BAL) fluid, (iii) the level of cytokines (IL-4, IL-5, and IL-13) in BAL fluid and lung homogenate, (iv) histopathological findings in the lung and nasal cavity, and (v) gene expression, using quantitative real-time polymerase chain reaction (qPCR).

## Materials and methods

### Animals

Forty female BALB/c mice, which were 4 to 6 weeks old and free of murine-specific pathogens, were purchased from Orient Bio (Seongnam, Korea). They were raised in a controlled environment, with a regular 12-hour light-dark cycle and unrestricted access to OVA-free food and water. All the mice that were used in this study were handled according to a protocol, which was approved by the INHA University Institutional Animal Care and Use Committee (INHA 150309–351).

### Sensitization and challenge with OVA

For the detailed experimental protocol, we followed the methods of Jang et al, 2016.[3] For the induction of allergic asthma, the mice were first sensitized with an intraperitoneal (i.p.) injection of 20 μg of OVA (Sigma-Aldrich, St. Louis, MO, USA) and 2.25 mg aluminum hydroxide gel (alum adjuvant; Thermo Fisher Scientific, Waltham, MA, USA) in 100 μl of sterile saline on days 0 and 14. After systemic sensitization, the mice were locally challenged by intranasal (i.n.) instillation of 500 μg of OVA into their nostrils from days 28, 29, and 30.

### Exposure to hypergravity

A gravitational force (G-force) simulator with two rotatory arms (50 cm long each), was developed, for the hypergravity experiments. When the arms are rotated, centrifugal force is exerted on the animal cage, which is suspended from the arms. When the arms rotate at a speed of 65 rpm, the mice in the cage are exposed to 5 G hypergravity. A high-resolution video camera, which was placed inside the cage, was used to evaluate whether the mice could move freely, and access food and water. In this experiment, the mice were exposed to simulated hypergravity for 30 consecutive days, during the entire period of sensitization and challenge. While exposed to hypergravity, mice were able to move freely within the cage and eat food and water. During this 30-day period, the G-force simulator was stopped once a day (for approximately 30 minutes), the vitality of the animals was checked, the intake of food and water was facilitated, and the i.p. sensitization or the i.n. challenge was performed. All mice were healthy during the 30-day exposure period, and were able to exercise vigorously during hypergravity exposures. No mice died during the experiment.

**Fig 1:**
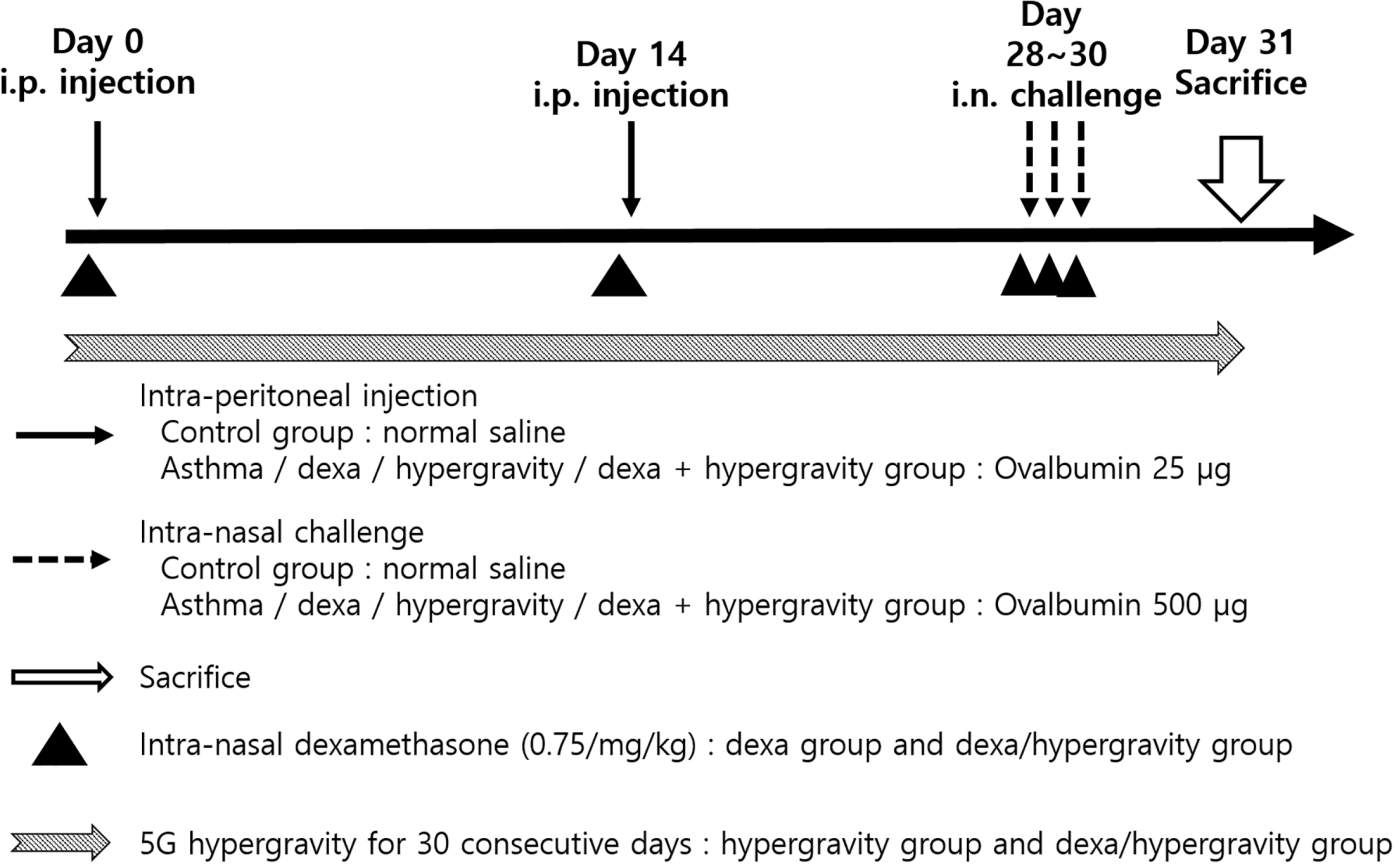
Study protocol for the induction of allergic asthma and rhinitis and/or exposure to hypergravity.

The mice were divided into five groups (n = 8 for each group). The mice in the “control group” were subjected to i.p. sensitization and i.n. challenge with sterile saline only. The mice in the “asthma group” underwent i.p. sensitization and i.n. challenge with OVA for the induction of allergic asthma. In the “dexa group”, the mice were administered 0.75 mg/kg of dexamethasone (Sigma-Aldrich, St. Louis, MO, USA) by i.n. instillation, 30 minutes before the i.p. sensitization and i.n. challenge. The mice in the control group, the asthma group, and the dexa group were all bred without being exposed to any rotatory stimulus (stationary control). The mice in the “hypergravity group” were continuously exposed to continuous hypergravity of 5 G for 30 days along with the induction of allergic asthma. Finally, the mice in the “dexa + hypergravity (combination therapy) group” were exposed to hypergravity of 5 G, and administered dexamethasone during the induction of allergic asthma in order to evaluate the effect of the combination therapy.(Fig 1)

### Collection and measurement of serum levels of total IgE and OVA-specific IgE

Twenty-four hours after the last i.n. instillation with saline or OVA, the G-force simulator was stopped, and the mice were sacrificed immediately by cervical dislocation (to minimize the recovery from the effect of hypergravity and the adaptation to normal gravity). Blood was collected from the abdominal aorta using an aortic puncture technique. Whole blood was centrifuged at 4°C for 30 minutes at 13,000 × *g*, and the supernatant (serum) was stored immediately at −80°C for future analysis.

For the analysis, the samples were diluted to 1:50. The serum levels of total IgE and OVA-specific IgE were measured using enzyme-linked immunosorbent assay (ELISA). Total IgE level was measured, and compared with a mouse IgE standard (BD Biosciences Pharmingen, San Diego, CA, USA). To determine the serum titers of OVA-specific IgE, the plate coated OVA 200 μg/mL with IgE-capture antibody was used, and the optical density was measured at 450 nm in accordance with the protocol provided by the manufacturer.

### Measurement of differential cell counts and cytokine levels in broncho-alveolar lavage (BAL) fluid

To collect BAL fluid, the trachea was first cannulated using polyethylene tubing, and then, the lung was lavaged with 800 μl of Hank’s balanced salt solution (HBSS; Thermo Fisher Scientific, Waltham, MA, USA) thrice repeatedly. The fluid was centrifuged at 4°C for 15 minutes at 3,000 *× g*, and the supernatant was stored immediately at −80°C for measuring the cytokine levels. The resulting pellet was suspended immediately in saline for cell counting.

Total cell numbers were determined in duplicate using a hemocytometer. Subsequently, an aliquot of 100−200 μl was centrifuged in a Cytospin 2 cytocentrifuge (Shandon Scientific, Pittsburgh, PA, USA). The differential cell counts of eosinophils, neutrophils, and lymphocytes were determined from the centrifuged preparations that were stained using the Diff-Quik stain kit (Sysmex Corp., Kobe, Japan) by counting 500 or more cells from each sample at 200x magnification.

The levels of cytokines in the supernatants of the BAL fluid were measured using individual ELISA kits (for IL-4 and IL-5, BD Biosciences Pharmingen, San Diego, CA, USA; for IL-13, R&D Systems, Minneapolis, MN, USA) according to the instructions of the manufacturers.

### Histopathological examination

Tissue specimens of the lung and nasal cavity were fixed in 4% paraformaldehyde solution for 24 hours. The lung tissues were washed with deionized water, and then, embedded in paraffin. The nasal tissues were also washed with deionized water, and then, immersed in EDTA solution for decalcification for 3 to 4 weeks. Then, they were embedded in paraffin in the same way. The tissue sections (of 3 μm thickness) were stained using hematoxylin and eosin solution (for the qualitative evaluation of histopathological changes), periodic acid-Schiff solution (for the staining of mucus) and Sirius red (for the evaluation of eosinophilic infiltration).

The number of eosinophils that had infiltrated into 1 mm^2^ of pulmonary parenchyma was counted in 20 random high-power fields (at 200× magnification). The number of eosinophils that had infiltrated into 1 mm^2^ of lamina papyracea was counted in the T1 area (the section, which is immediately posterior to the upper incisors), for each sample, in 10 high-power fields (×400 magnification). The histopathological examinations were performed, and the eosinophils in the tissue specimens were counted by two impartial, blinded researchers.

### Quantitative real-time PCR

Immediately after harvesting whole lung tissue from each mouse, the tissue samples were frozen in liquid nitrogen. Total RNA was isolated using TRIzol reagent (Invitrogen Life Technologies, Waltham, MA, USA) according to the protocol that was recommended by the manufacturer. Complementary DNA was synthesized from 1 μg of total RNA by using the PrimeScript RT reagent kit (Takara Bio Inc., Shiga, Japan).

Real-time PCR was performed in duplicate using the StepOnePlus^™^ Real-Time PCR System (Applied Biosystems Inc., Carlsbad, CA, USA). Primer annealing temperatures and number of cycling were as follows: 95°C for 10 minutes, 40 cycles of 95°C for 15 seconds and 60°C for 1 minute. For identifying amount of GAPDH, we first checked gel band of GAPDH of RT-PCR. After comparing band density between groups, we could find that the amount of GAPDH mRNA is the stable between the 5 groups of mice. Therefore, we proceeded real-time PCR. The sequence features of the target gene were identified from the NCBI website, and the primers were designed using Primer 3.0 software (the sequences of the primers are shown in Table 1, except those of the primers for IL-4, IL-5, and IL-13). The primers for IL-4, IL-5, and IL-13 were purchased from QuantiTect Primer Assays (QIAGEN, Venlo, The Netherlands). In order to calculate the efficiency of the amplification, the relative quantity of each target gene was normalized to that of the housekeeping gene *GAPDH* using comparative 2^−ΔΔ*CT*^ method.

**Table 1:** Primer sequences for real-time polymerase chain reaction.

Gene		Primer Sequence
*Casp3*	Forward	5′-TGG GCC TGA AAT ACC AAG TC-3′
	Reverse	5′-AAA TGA CCC CTT CAT CAC CA-3′
*COX2*	Forward	5′-TTC GGG AGC ACA ACA GAG TG-3′
	Reverse	5′-TAA CCG CTC AGG TGT TGC AC-3′
*EC-SOD*	Forward	5′-GTG TCC CAA GAC AAT C-3′
	Reverse	5′-GTG CTA TGG GGA CAG G-3′
*HO-1*	Forward	5′-CCT CAC TGG CAG GAA ATC ATC-3′
	Reverse	5′-CCT CGT GGA GAC GCT TTA CAT A-3′
*Hif-1α*	Forward	5′-ACC TTC ATC GGA AAC TCC AAA G-3′
	Reverse	5′-CTG TTA GGC TGG GAA AAG TTA GG-3′
*VEGF*	Forward	5′-CTT GTT CAG AGC GGA GAA AGC-3′
	Reverse	5′-ACA TCT GCA AGT ACG TTC GTT-3′
*GAPDH*	Forward	5′-GCA CAG TCA AGG CCG AGA AT-3′
	Reverse	5′-GCC TTC TCC ATG GTG GTG AA-3′

### Statistical analyses

The nonparametric tests, such as the Kruskal-Wallis test and the Mann-Whitney U test were performed to compare the titers of total IgE and OVA-specific IgE, the number of inflammatory cells, the levels of cytokines in the BAL fluid, the number of eosinophils that infiltrated into the pulmonary parenchyma and the nasal cavity, and the degree of gene expression among the groups. A *p* value < 0.05 was considered statistically significant.

## Results

### Combination of dexamethasone and hypergravity significantly decreased serum IgE levels

**Fig 2:**
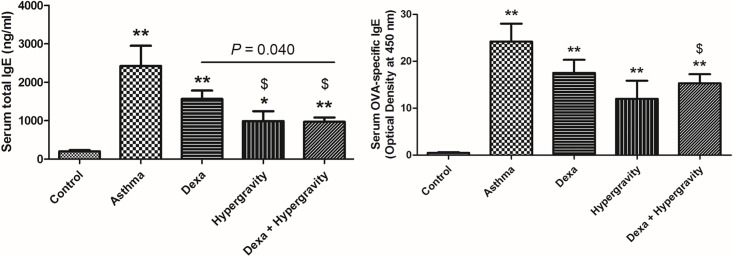
Serum levels of total IgE and ovalbumin (OVA)-specific IgE. * Significant difference from the control group, *p* < 0.05; ** significant difference from the control group, *p* < 0.01; $ significant difference from the asthma group, *p* < 0.05 (the Kruskal-Wallis and Mann-Whitney U tests).

The serum levels of total IgE and OVA-specific IgE increased markedly in the asthma group compared with the control group (*p* < 0.01). After 4 weeks of continuous exposure to the rotatory G-force, the hypergravity group exhibited significantly decreased serum total IgE levels compared with the asthma group (*p* < 0.05). This decrease in serum IgE levels in the hypergravity group was quite comparable to that in the dexa group. Furthermore, the dexa/hypergravity (combination therapy) group exhibited significantly more decreased serum total IgE levels compared with the dexa group (*p* = 0.040). Analysis revealed a significant decrease in the OVA-specific IgE levels in the dexa/hypersensitivity group compared with the asthma group. (*p* < 0.05) (Fig 2).

### Combination of dexamethasone and hypergravity resulted in significantly more decrease in the number of eosinophils in the BAL fluid compared with monotherapy

**Fig 3:**
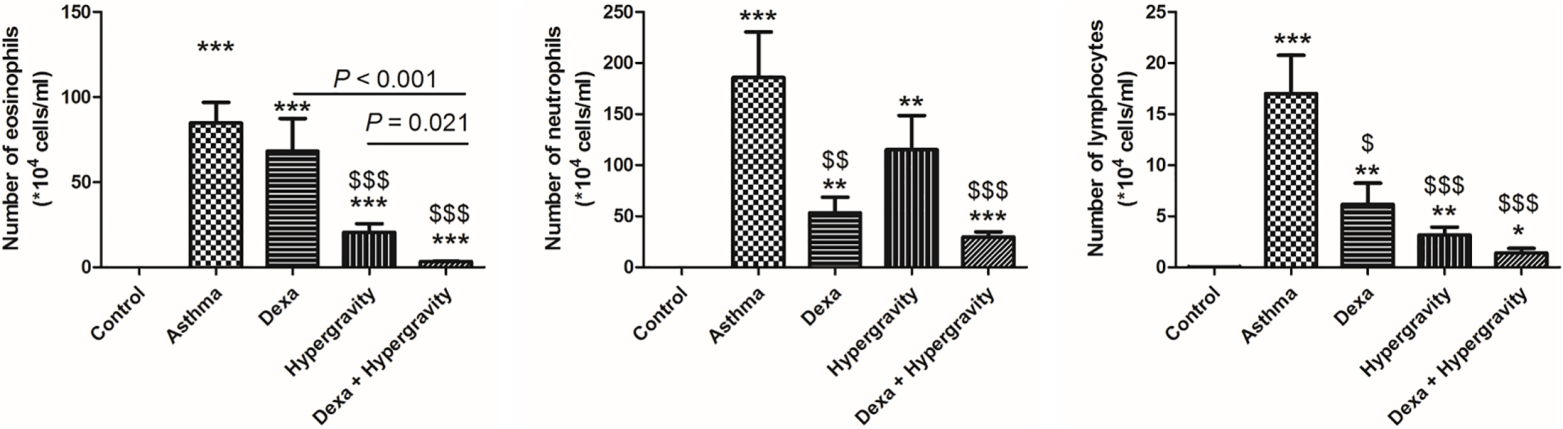
Number of eosinophils, neutrophils, and lymphocytes in the broncho-alveolar lavage fluid. * Significant difference from the control group, *p* < 0.05; ** significant difference from the control group, *p* < 0.01; *** significant difference from the control group, *p* < 0.001; $ significant difference from the asthma group, *p* < 0.05; $ $ significant difference from the asthma group, *p* < 0.01; $ $ $ significant difference from the asthma group, *p* < 0.001 (the Kruskal-Wallis and Mann-Whitney U tests).

Compared with the control group, the number of all inflammatory cells, including eosinophils, neutrophils, and lymphocytes in the BAL fluid increased significantly in the asthma group (*p* < 0.001). Compared with the monotherapy groups (the dexa group and the hypergravity group), the dexa/hypergravity combination group showed a tendency of decrease in the number of inflammatory cells in the BAL fluid, with a statistically significant difference in the number of eosinophils (*p* < 0.05) (Fig 3).

### Hypergravity significantly decreased gene expression and secretion of Th2 cytokines

The asthma group showed a statistically significant upregulation in gene expression of IL-4 and IL-5, evaluated using the homogenate of lung parenchyma). They also showed an increase in the secretion (evaluated using the BAL fluid) of Th2 cytokines, including IL-4, IL-5, and IL-13 compared with the control group (*p* < 0.05).

**Fig 4:**
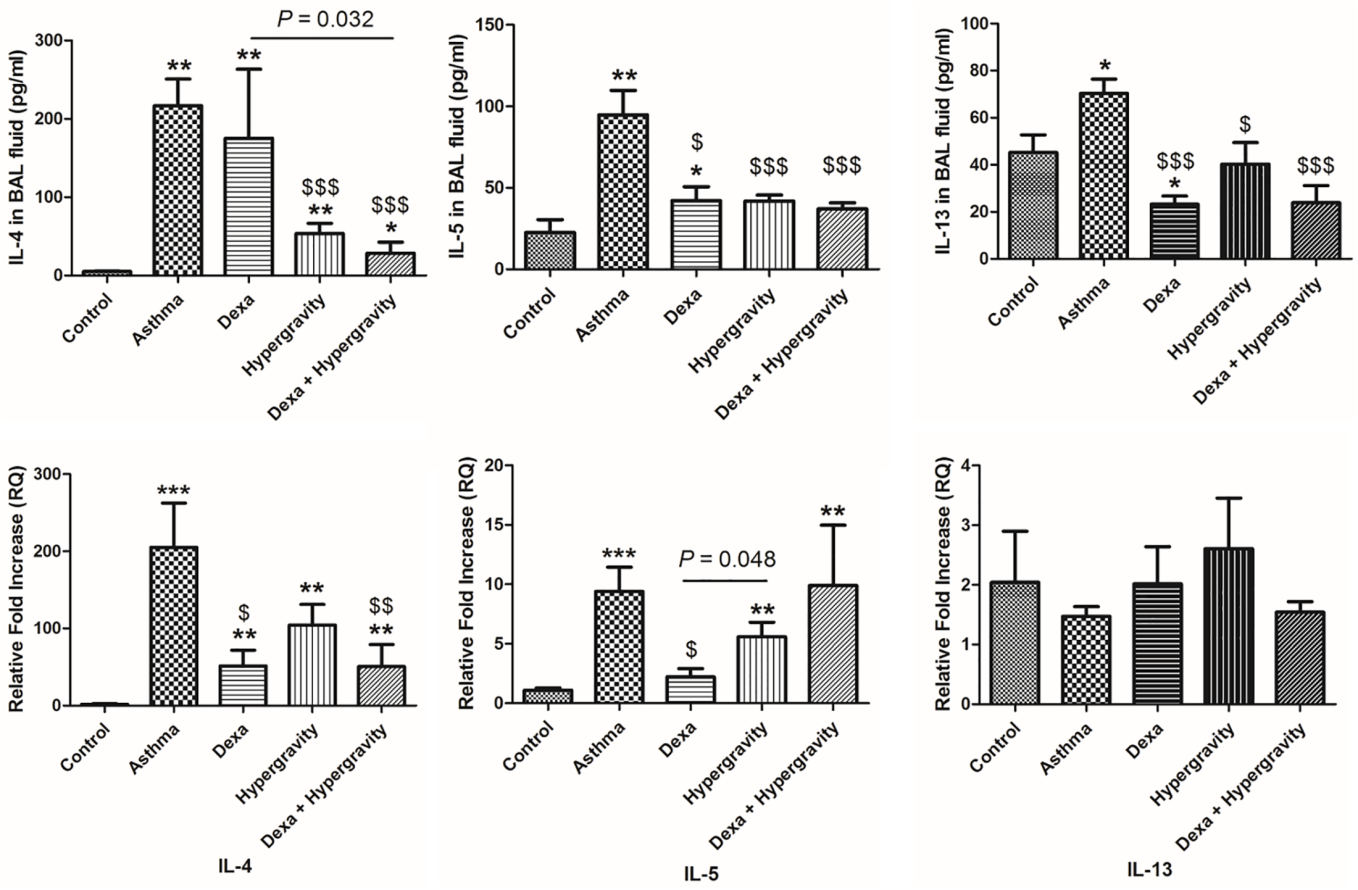
Levels of IL-4, IL-5, and IL-13 in the broncho-alveolar lavage fluid (upper column) and expression of IL-4, IL-5, and IL-13 gene in the lung homogenate, analyzed using PCR (lower column). * Significant difference from the control group, *p* < 0.05; ** significant difference from the control group, *p* < 0.01; *** significant difference from the control group, *p* < 0.001; $ significant difference from the asthma group, *p* < 0.05; $ $ significant difference from the asthma group, *p* < 0.01; $ $ $ significant difference from the asthma group, *p* < 0.001 (the Kruskal-Wallis and Mann-Whitney U tests).

In the dexa/hypergravity group, IL-4, IL-5 and IL-13 secretion in the BAL fluid decreased statistically significantly (*p* < 0.001) compared with the asthma group. In particular, IL-4 showed a more significant decrease in dexa/hypergravity group than the dexa group (*p* = 0.032, Fig 4).

### Dexa/hypergravity group demonstrated more significant decrease in eosinophilic infiltration into the lung and nasal cavity

**Fig 5:**
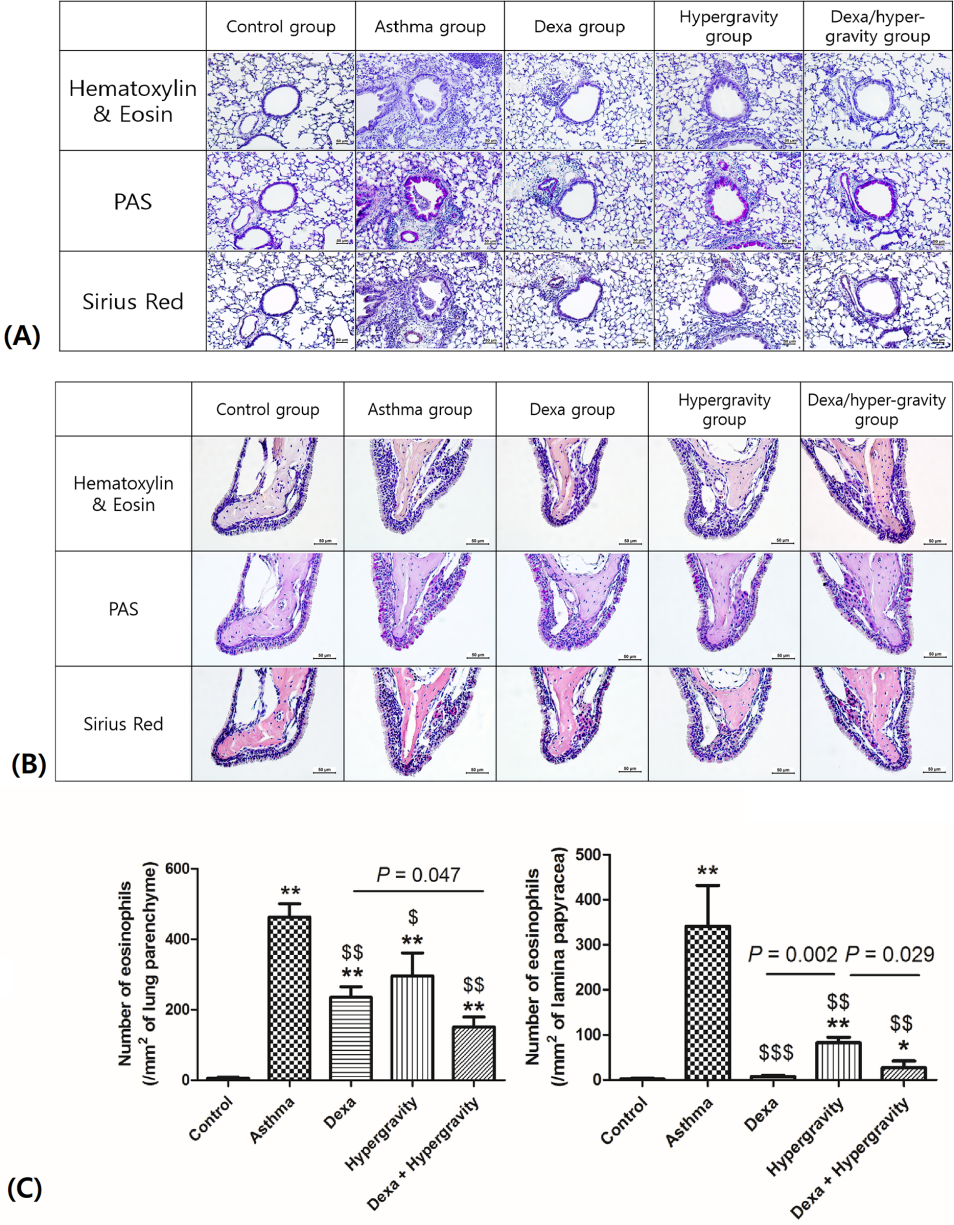
Histological observations of the lung and nasal cavity (upper column, Figs 5a_1 and 5a_2), and the number of infiltrated eosinophils into 1 mm^2^ of lung parenchyma and lamina papyracea of the nasal cavity (lower column, Fig 5b). * Significant difference from the control group, *p* < 0.05; ** significant difference from the control group, *p* < 0.01; $ significant difference from the asthma group, *p* < 0.05; $ $ significant difference from the asthma group, *p* < 0.01; $ $ $ significant difference from the asthma group *p* < 0.001 (the Kruskal-Wallis and Mann-Whitney U tests).

The asthma group showed a significantly increased infiltration of eosinophils into the lung parenchyma and lamina propria of the nasal cavity compared with the control group (*p* < 0.01). The monotherapy groups (the dexa group and the hypergravity group) showed a statistically significant reduction in eosinophilic infiltration compared with the asthma group (*p* < 0.01). Finally, the dexa/hypergravity combination group showed a tendency of a more significant decrease in eosinophilic infiltration compared with the monotherapy group, and this tendency was statistically significant (the dexa group versus the dexa/hypergravity group in the lung, *p* = 0.047; the hypergravity group versus the dexa/hypergravity group in the nasal cavity, *p* = 0.029) (Fig 5).

### Expression of extracellular superoxide dismutase (EC-SOD) was significantly up-regulated in the hypergravity and combination therapy groups

**Fig 6:**
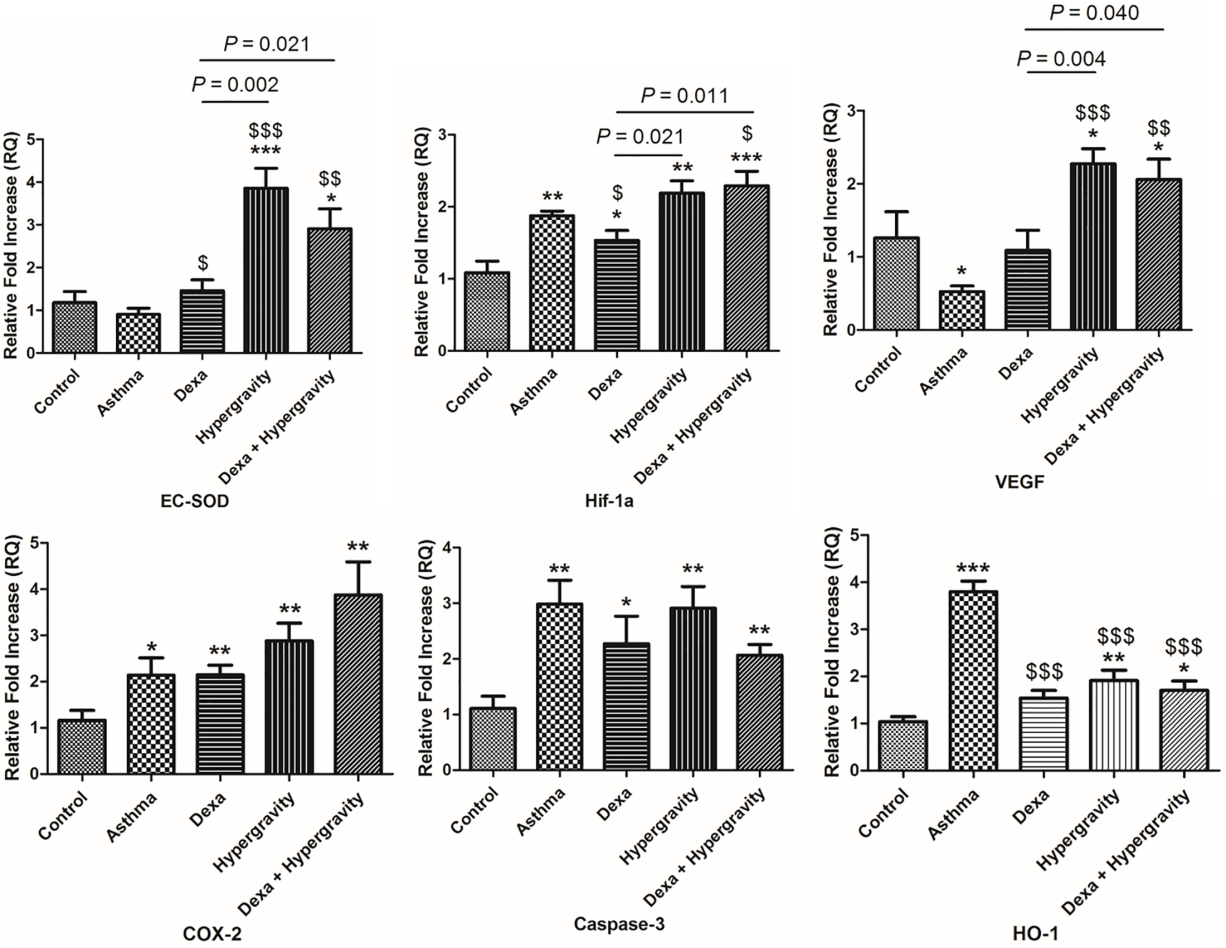
Expression of extracellular superoxide dismutase (*EC-SOD*), Hypoxia-inducible factor-1α (*Hif-1α*), Vascular endothelial growth factor (*VEGF*), Cyclo-oxygenase-2 (*COX-2*), caspase-3, and heme oxygenase-1 (*HO-1*) genes, evaluated by real-time PCR. * Significant difference from the control group, *p* < 0.05; ** significant difference from the control group, *p* < 0.01; *** significant difference from the control group, *p* < 0.001; $ significant difference from the asthma group, *p* < 0.05; $ $ significant difference from the asthma group, *p* < 0.01; $ $ $ significant difference from the asthma group, *p* < 0.001 (the Kruskal-Wallis and Mann-Whitney U tests).

A significant increase in *EC-SOD* gene expression was observed in the hypergravity and dexa/hypergravity groups compared with the asthma group (*p* < 0.01) and the dexa group (*p* < 0.05). It was also found that the hypergravity group and the dexa/hypergravity group showed a significantly higher expression of the Hypoxia-inducible factor 1a (*Hif-1α*) and Vascular endothelial growth factor (*VEGF*) genes than the dexa group in (*p* < 0.05). On the other hand, there was a tendency of greater increase in the expression of Cyclo-oxygenase 2 (*COX-2*) in the dexa/hypergravity group than the other groups; however, it was not statistically significant. In case of the expression of the caspase-3 gene, there was no significant difference among the groups. Finally, the expression of the heme oxygenase-1 (*HO-1*) gene was significantly suppressed in all the treatment groups compared with the asthma group (*p* < 0.001); however, no significant difference was observed among the treatment groups (Fig 6).

## Discussion

After 4 weeks of exposure to 5G of rotatory G-force, the mice in the hypergravity group showed a statistically significant decrease in serum total IgE levels compared with the mice in the asthma group. This reduction in IgE levels was comparable to that of the dexa group (in fact, there were no statistically significant differences between the hypergravity group and the dexa group). These findings are in line with our previously conducted studies.[10] Guéguinou et al. reported that when animals were exposed to 2 G hypergravity for 3 weeks, serum IgG levels increased more than 2-fold, and IgA levels increased by 30%.[8] However, to the best of our literature review, it is the first time that we have observed changes in IgE levels of mice after their exposure to hypergravity. The dexa group, the hypergravity group and the dexa/hypergravity combination therapy group showed a tendency of decrease in the OVA-specific IgE levels also, compared with the asthma group (however, there was no statistical significance). Based on the results of the present study, it could be suggested that hypergravity is at least as efficacious as medical treatments, such as the treatment with dexamethasone, in reducing serum IgE levels. Our previous studies have shown that hypergravity has a hormetic effect in terms of immunology.[10] In this study, we proposed that this hormesis could be as efficacious as medication, and that if we combine these two treatment modalities, they would have an additive or synergistic effect. In this way, we suggested that this combination therapy could be a potential next-generation adjunctive treatment for allergic disorders.

After 4 weeks of exposure to 5 G of rotatory G-force, the mice in the hypergravity group showed a decrease in the number of inflammatory cells (eosinophils, neutrophils, and lymphocytes) in the BAL fluid compared with those in the asthma group. These results are also consistent with our previous findings.[10] In our present study, we further found that, when dexamethasone and hypergravity were combined, the number of eosinophils tended to decrease more markedly in the combination therapy group than in the monotherapy group. In the combination therapy group, the number of neutrophils and lymphocytes also decreased; however, it was not statistically significant. Based on these findings, we plan to perform *in vitro* studies to evaluate the type of immune cells that are more affected by hypergravity.

We aimed to study the expression of the Th2 cytokine (IL-4, IL-5, and IL-13) genes, which are implicated in allergic reactions, and changes in the actual secretion of the cytokines. Therefore, we performed quantitative real-time PCR using lung homogenate and ELISA using BAL fluid. In our study, gene expression of IL-4, IL-5, and IL-13 did not show a statistically significant decrease (*p* > 0.05) in the hypergravity group, compared to the asthma group. We need to compare this result with our previously published findings. In our previous study, the hypergravity group showed a statistically significant decrease in IL-4 gene expression when compared to the asthmatic group, but this was at the same level as the rotary stimulus control group. In the case of IL-5, no significant difference was observed between asthma group and hypergravity group (IL-13 is not included in our previous study).[10] In contrast, the dexa/hypergravity group showed a statistically significant decrease in IL-4 gene expression when compared to the asthma group. Therefore, dexa/hypergravity combination treatment has at least the effect of decreasing the gene expression and thus the secretion of IL-4 in the BAL fluid. Pecaut et al. exposed C57BL/6 mice continuously to hypergravity of 2 G or 3 G for 3 consecutive weeks, and observed changes in the concentrations of IL-4 and IL-5 in splenic leukocytes. The concentration of these cytokines began to increase at the beginning of the experiment, and then, decreased. After 20 days, the levels of the cytokines were lower than normal.[3] These results also coincide with those of our previous research study.[10] Although not statistically significant, gene expression and cytokine secretion, especially of IL-4 and IL-13, decreased further in the combination therapy groups. To determine if this phenomenon is in fact significant, we should plan further studies on a larger number of experimental animals. On the other hand, Guéguinou et al. measured the titer of IL-4 and IL-5 in serum after 3 weeks of exposure to 2G or 3G of hypergravity. They observed a decrease of IL-4 as G force increased but no effect on IL-5 production. Perhaps this would be due to differences in experimental animal strain (C57BL/6J male versus BALB/c female in our study), experimental protocol (intensity and duration of hypergravity stimulation). To test this hypothesis, we have plans to carry out additional experiments at 2G or 3G of hypergravity.

We assessed the degree of eosinophil infiltration into the lung parenchyma and nasal cavity, qualitatively and quantitatively. We found that eosinophilic infiltration was significantly reduced in the hypergravity group compared with the asthma group. Furthermore, the hypergravity group and the dexa group showed a similar degree of reduction in inflammatory cell infiltration. Eosinophilic infiltration, in particular, tended to decrease more in the combination therapy group than in the monotherapy group (the dexa and hypergravity groups), and statistical significance was also found in the nasal cavity.

To investigate the mechanism of this anti-allergic effect of hypergravity, and the effect of its combination with dexamethasone, we performed quantitative real-time PCR for several candidate genes. EC-SOD performs an antioxidant function by removing reactive oxygen free radicals (such as superoxide). Research on the link between these antioxidant functions and anti-allergic effects has been continuing. Kwon et al. reported that the allergic responses were improved when EC-SOD was administered to mice with allergic asthma.[11] In addition, severe allergic asthma was induced in *EC-SOD* knockout mice.[11] Taken together, these results suggest that increasing the expression of *EC-SOD* can be a good strategy for anti-allergic treatments. In the hypergravity group and the combination therapy group, *EC-SOD* expression was significantly upregulated. Therefore, this supports the hypothesis that the mechanism of the anti-allergic effects of hypergravity is related to antioxidative effects.In addition, the combination therapy group showed statistically significant, better anti-allergic effect than the dexamethasone monotherapy group.

HIF consists of two subunits, α and β. While β subunit is secreted constantly, the secretion of α subunit is regulated according to the condition of the body. HIF is activated in hypoxic environments, and induces the increase in the levels of various inflammatory cytokines, chemokines, adhesion molecules, and VEGF.[12] Therefore, HIF plays a role in amplifying systemic inflammation in a hypoxic environment. Hypergravity, which was used in our experiments can be considered to generate a type of hypoxic microenvironment. In a study of lung perfusion, which was assessed using quantitative SPECT after the exposure of healthy volunteers to hypergravity, Ax et al. suggested that arterial oxygen desaturation, which is due to the redistribution of blood flow and ventilation, occurs because of hypergravity.[13] If the mechanisms of the anti-allergic of hypergravity were related to HIF-1 inhibition, the expression of this gene would have been suppressed. In fact, when the siRNA that inhibits *HIF-1* expression was administered to experimental animals with allergic diseases, the progress of the disease was controlled.[14,15] In our former studies, we also found that Hif-1 and VEGF levels decreased after the treatment of experimental animals with allergic asthma, with benzaldehyde.[16] However, in this experiment, the expression of these genes remained increased because of the potential hypoxic effect of hypergravity. Therefore, it can be presumed that the anti-allergic effect of hypergravity is not related to the regulation of these genes.

The expression of *COX-2* tended to be increased in the hypergravity group and the combination therapy group compared with the asthma group, although without statistical significance. The induction of cyclooxygenase by gravity stimuli is known to play an important role in bone tissue homeostasis.[17,18] Recently, it has been reported that mechanical stress caused by hypergravity can induce *COX-2* expression in bone tissues as well as in other normal tissues. Oshima et al. analyzed the expression pattern of *COX-2* after exposing tissues of various organs, such as lung, brain, and heart to hypergravity of 2 G or 3 G for 4 hours. The expression of COX-2 mRNA in these tissues significantly increased compared with tissues exposed to normal gravity. In order to determine whether the *COX-2*-dependent expression of *Hif-1α* or *VEGF* is involved, wild type mice and *COX-2* knockout mice were exposed to hypergravity. The expression of *HIF-1α* and *VEGF* was not upregulated in the *COX-2* knockout mice in contrast to the wild type mice.[19] We need to consider the relationship between cyclooxygenase and allergic inflammation. Meng et al. reported that the expression of *COX-2* decreased, when epigallocatechin (candidate therapeutic agent for allergy) was administered to mice with OVA-induced allergic rhinitis.[20] However, since *COX-2* expression was not changed because of hypergravity in our study, we can speculate that the mechanism of the anti-allergic effect of hypergravity is independent of *COX-2*.

There was no significant association between hypergravity exposure and *Casp3* expression. Therefore, the anti-allergic effect of the combination therapy with hypergravity and dexamethasone appears to be independent of *Casp3* expression. This is consistent with our previous findings.[10]

HO-1 has been known to play a protective role against airway inflammation and hyperresponsiveness by inhibiting the degranulation of mast cells and synthesis of Th2 cytokines.[16,21] According to previous studies, the experimental animals, which were sensitized with OVA showed increased expression of *HO-1*, which was significantly suppressed after treatment.[22] Elhini et al. also reported that the expression of *HO-1* had also increased significantly in patients with persistent allergic rhinitis.[23] Therefore, the suppression of *HO-1* expression is expected to be an excellent strategy for anti-allergic treatment.[24,25] In our present study also, the hypergravity group exhibited the significantly suppressed expression of *HO-1*, which was quite comparable with that of the dexa group.

This study was based on a fixed protocol of exposure to hypergravity of 5 G for 4 weeks. Probably, the changes in the acute and chronic adaptations that occurred *in vivo* during 4 weeks after the exposure to hypergravity could be different. Therefore, in order to expand the results of this study, it would be possible to obtain additional results that are more meaningful, if studies are conducted for different periods in each group of experimental animals. In order to investigate the changes in gene expression further after hypergravity exposure and combination therapy, a method of screening all genes using microarrays can be considered. Finally, by exposing the animals to hypergravity of varying magnitude ranging from 1.1 G to 10 G as well as 5 G, and by uniformly comparing and analyzing the changes in each case, we could find the optimal magnitude of hypergravity with the highest safety and optimal anti-allergic effect.

Since it is extremely rare for people to be exposed to hypergravity for such a prolonged period of time, one could think that results of these studies are actually clinically meaningless. However, recent studies have shown that varied species of animals have different sensitivities to hypergravity, depending on their body weight. In other words, mice with extremely low body weight can survive longer even at hypergravity of 9G, while humans do not survive for lengthy periods at hypergravity as high as 2.7G.[26] In addition, as shown in our previous study, mice survived for 4 weeks in a hypergravity of about 5G.[10] Therefore, it can be inferred that exposure to hypergravity for a longer period is required in experimental animals, in order to reproduce similar changes in human beings.

In this experiment, mice exposed to hypergravity by centrifuge were probably exposed to various stresses such as rotational stimuli, vibration, and different handling besides increased gravity. And these stimuli may influence cytokine secretion and gene expression in experimental animals. Therefore, it is most desirable to use a rotatory control group, that is, a centrifuge rotating at a very low speed, to set an environment in which gravity itself is normal and other stimuli were given such as rotational stimulation. In fact, our previous study also established a rotatory control group.[10] It has shown that hypergravity itself has an anti-allergic effect, after excluding the effects of other nonspecific stimuli. Therefore, in this experiment, we used only the static control and compared the effect of the single treatment group and the combined treatment group.

Since hypergravity stimuli are also a kind of ‘stress’, we can not rule out the possibility that corticosterone, secreted after hypergravity stimulation, could have an anti-allergic effect. Previous studies have shown that stress response depends on the model used to modify gravity force. While centrifugation, used to increase gravity force, increases corticosterone concentration[8,27], anti-orthostatic tail suspension of rodents, used to reduce gravity constraint, did not affect the level of this hormone.[28,29] However, in our previous study we have already demonstrated that the hypergravity group had a greater anti-allergic effect when compared to the rotatory control group. We could infer that there would be no significant difference between the rotatory control group and the hypergravity group if the corticosterone caused by stress was the cause. Therefore, we hypothesized that the anti-allergic effect of hypergravity may have other causes besides the action of corticosterone. We expect that further research will provide additional interesting results if we analyze this in future studies.

In conclusion, hypergravity has anti-allergic effects that are comparable to those of drug treatment in the murine model of OVA-induced allergic asthma and rhinitis. Combining drug therapy with hypergravity may be a promising therapeutic strategy in the future. Finally, the mechanism of this anti-allergic effect of hypergravity could be related to the up-regulation of the antioxidant gene *EC-SOD*.
